# ABCC6- a new player in cellular cholesterol and lipoprotein metabolism?

**DOI:** 10.1186/1476-511X-13-118

**Published:** 2014-07-27

**Authors:** Patricia Kuzaj, Joachim Kuhn, Mareike Dabisch-Ruthe, Isabel Faust, Christian Götting, Cornelius Knabbe, Doris Hendig

**Affiliations:** 1 Herz- und Diabeteszentrum NRW, Institut für Laboratoriums- und Transfusionsmedizin, Universitätsklinik der Ruhr-Universität Bochum, Georgstraße 11, 32 545 Bad Oeynhausen, Germany; 2 MVZ Labor Limbach Nürnberg, Lina-Ammon-Straße 28, 90471 Nürnberg, Germany

**Keywords:** Pseudoxanthoma elasticum, ABC transporter, ABCC6, Cholesterol biosynthesis, Atherosclerosis, HMG CoA reductase, SREBP2, PCSK9, LDLR, APOE

## Abstract

**Background:**

Dysregulations in cholesterol and lipid metabolism have been linked to human diseases like hypercholesterolemia, atherosclerosis or the metabolic syndrome. Many ABC transporters are involved in trafficking of metabolites derived from these pathways. *Pseudoxanthoma elasticum* (PXE), an autosomal-recessive disease caused by ABCC6 mutations, is characterized by atherogenesis and soft tissue calcification.

**Methods:**

In this study we investigated the regulation of cholesterol biosynthesis in human dermal fibroblasts from PXE patients and healthy controls.

**Results:**

Gene expression analysis of 84 targets indicated dysregulations in cholesterol metabolism in PXE fibroblasts. Transcript levels of ABCC6 were strongly increased in lipoprotein-deficient serum (LPDS) and under serum starvation in healthy controls. For the first time, increased HMG CoA reductase activities were found in PXE fibroblasts. We further observed strongly elevated transcript and protein levels for the proprotein convertase subtilisin/kexin type 9 (PCSK9), as well as a significant reduction in APOE mRNA expression in PXE.

**Conclusion:**

Increased cholesterol biosynthesis, elevated PCSK9 levels and reduced APOE mRNA expression newly found in PXE fibroblasts could enforce atherogenesis and cardiovascular risk in PXE patients. Moreover, the increase in ABCC6 expression accompanied by the induction of cholesterol biosynthesis supposes a functional role for ABCC6 in human lipoprotein and cholesterol homeostasis.

## Background

ATP Binding Cassette (ABC) proteins are important transporters for the delivery of hydrophobic compounds across cellular membranes [1]. To date, 48 human transporters have been described which can be classified into 7 subgroups [2]. Many ABC transporters are involved in trafficking of metabolites derived from lipid or cholesterol biosynthesis, e.g. sterols, bile acids, phospholipids, or sphingolipids [3]. Moreover, genetic mutations in ABC transporters have been linked to various human diseases [2,3], like *Dubin–Johnson syndrome* (ABCC2), *Tangier disease* (ABCA1), or *Pseudoxanthoma elasticum* (ABCC6).

In contrast to other human transmembrane transporters, the characteristics and substrate spectra of which have already been explored, the function and physiological role of ABCC6 is still unclear [4]. *Pseudoxanthoma elasticum* (PXE) is an autosomal recessive disorder with an estimated prevalence of 1: 25.000- 50.000 [5]. To date, up to 350 causative genetic mutations have been found in ABCC6 [6].

PXE is characterized by soft tissue calcification affecting the skin, eyes and cardiovascular system [7]. Morphologically, mineralization occurs on elastic fibers which show increased degradation in PXE patients, in addition to abnormalities in collagen fibril assembly [8] and accumulation of proteoglycans [9]. Patients suffer from premature atherosclerosis, reduction in skin elasticity and visual detractions (angioid streaks, peau d’orange) [10]. Yellowish papules, marking flexural body sites, appear during the course of the disease [11]. Typically, xanthomas are characterized by cholesterolester accumulations in dermal foam cells [12], where cutaneous lesions of PXE derived from elastin calcification and fragmented fiber deposits [13]. The involvement of cholesterol or lipid depositions in PXE papule development is not clear. However, lipoproteins like the low density lipoprotein (LDL) have been examined for their capacity to bind to elastin, which increases under atherosclerotic conditions [14,15].

ABCC6 is primarily expressed in the human liver and kidney, and to a lesser extent has been found in the skin, neural retina and vessel walls [16]. Moreover, Beck *et al.* detected ABCC6 mRNA in murine intestine, colon, brain, and eye [17]. Human dermal fibroblasts served as an appropriate model for soft tissue calcification in recent studies [7,18,19]. In addition to human skin fibroblasts, Abcc6-deficient (Abcc6 ^−^/^−^) mouse models were established for the examination of PXE pathogenesis [20,21].

Regarding the functionality and physiological role of ABCC6, essential knowledge is still missing. Several studies have been carried out to elucidate substrate specifications of ABCC6 *in vitro*, demonstrating the transport of glutathione conjugates [10]. However, *in vitro* experiments and investigations of Abcc6 in mice showed no transport activity for vitamin K3-glutathione conjugates or adenosine [22,23], rejecting previous ideas for its potential functionality [24,25].

About twenty ABC transporters are involved in the carriage of compounds derived from lipid- or cholesterol metabolism and are important for reverse cholesterol transport (RCT) and phospholipid- and cholesterol efflux [3,26]. Voloshyna and Reiss summarized the functional role of ABCA1, ABCG1, ABCG4, ABCA5 and ABCA7 transporters in high-density lipoprotein (HDL)-mediated RCT, as well as ABCG5, ABCG8, ABCB4 and ABCB11 for biliary lipid secretion within the scope of atheroprotection [26]. Additionally, members of the ABCC subgroup, like ABCC1, ABCC2 and ABCC3, are further needed for bile acid and bilirubin efflux [3]. Studies have described genetic mutations in ABCC6 to be associated with variations in quantitative plasma lipoproteins [27], low HDL-C and/or coronary heart disease (CHD) risk [28]. Alterations in lipoprotein composition with lowered plasma HDL cholesterol levels and hypertriglyceridemia were found in plasma samples of PXE patients [29]. Furthermore, experiments in Abcc6 ^−^/^−^ mice showed a 25% reduction in plasma HDL cholesterol [20], confirming the potential role of ABCC6 in lipid homeostasis as described before [30]. Recently, Guo *et al.* demonstrated that atorvastatin counteracts soft tissue mineralization in Abcc6-deficient mice [31]. Statins are widely used to inhibit HMG CoA reductase activity, the rate-limiting step in cholesterol biosynthesis, to reduce plasma low-density lipoprotein cholesterol (LDL-C) levels and CHD risk [32]. Oxidized-LDL fractions have been described as the major stimuli for plaque formation and atherosclerotic development [33], as well as for angiogenesis [34]. In addition to statin treatments, inhibition of the proprotein convertase subtilisin/kexin type 9 (PCSK9) is under recent examination to lower circulating LDL levels, while PCSK9 negatively regulates the low-density lipoprotein receptor (LDLR) by promoting its lysosomal degradation [35].

Here, we describe for the first time HMG CoA reductase activity measurements in human dermal fibroblasts derived from PXE patients and healthy controls, as well as for ABCC6-silenced cells. Moreover, we carried out gene expression profiling of 84 targets involved in cholesterol biosynthesis and lipoprotein signaling in human dermal fibroblasts cultivated without fetal calf serum (−FCS) for 24 h. Significant alterations were further investigated under different cell culture conditions, using 10% fetal calf serum, 10% lipoprotein-deficient serum (LPDS) and serum starvation (−FCS) to stimulate HMG CoA reductase activity. Our results indicate that functional loss or dysfunction of ABCC6 in PXE dermal fibroblasts is significantly associated with alterations in cellular cholesterol metabolism and lipoprotein assembly.

## Materials and methods

### Cell culture

Primary human dermal fibroblasts from four PXE patients were isolated and specified as described previously [18]. None of the investigated patients was reported to suffer from dyslipidemia. Dermal fibroblasts from four apparently healthy controls were purchased from Promocell (Heidelberg, Germany), Genlantis (San Diego, USA), Cambrex (Walkersville, USA) and Coriell Institute for Medical Research (Camden, USA). All cells were isolated and characterized by standard methods. The study was approved by the ethics commission of the Ruhr University of Bochum Faculty of Medicine, located in Bad Oeynhausen. All patients provided their written informed consent to participate in the study. Characterization of human dermal fibroblasts is given in Additional file 1: Table S1. Cultivation was performed in Dulbecco’s modified essential medium (DMEM, Gibco) containing 10% fetal calf serum (PAN Biotech, Aidenbach, Germany), 1% L-glutamine (200 mM) and 1% antibiotic/antimycotic solution without phenol red, whereupon cells were subcultured every 4–5 days (1:3) as they reached confluence.

For experiments cells were grown for 24 h in 10% FCS (177 cells/mm^2^, BD Falcon), washed twice with phosphate-buffered saline (PBS; Gibco), and replaced with either 10% fetal calf serum (FCS), lipoprotein-deficient FCS (LPDS) [36], or without serum for additional 24 h. Cells reached approx. 70- 80% of confluence after stated time of growth. Biological samples were prepared in triplicates using passages 7–10. Cells harvested for RNA isolation and protein lysis were treated as described before [37,38].

### Gene silencing of ABCC6 using small-interfering RNA

Lipofectamine 2000 reagent (Invitrogen- Life technologies, Darmstadt, Germany) was used to deliver ABCC6-specific small-interfering RNA (siRNA-ID 106395) and FAM labeled scrambled control siRNA oligonucleotides (Ambion- Life technologies, Darmstadt, Germany) to dermal fibroblasts of healthy controls during reverse transfection, in a total siRNA-concentration of 40 nM. No antibiotic/antimycotic solution was used during the first 12 h. Cell culture medium was replaced with fresh media 12 h post-transfection, whereupon transfection efficiencies were examined by fluorescence microscopy of FAM labeled controls. Cells were cultivated for additional 48 h in 10% FCS, followed by 24 h cultivation in either 10% FCS, 10% lipoprotein deficient serum (LPDS), or without FCS.

### RT^2^ Profiler PCR Array

A broad gene expression analysis was performed covering 84 target genes involved in lipoprotein signaling and cholesterol metabolism by using RT^2^ Profiler PCR Array (Qiagen, Hilden, Germany, PAHS 080ZA). PCR array was performed with cells cultivated for 24 h without FCS, and 3 biological replicates were pooled for array analysis. RNA was isolated as described before [37], whereupon 400 ng were used for cDNA-synthesis (RT^2^ First Strand Kit; Qiagen, Hilden, Germany). Real-Time PCR was performed using LightCycler® 480 (Roche, Penzberg, Germany) and RT^2^ SYBR Green qPCR reaction mixture (Qiagen, Hilden, Germany). Reaction mixture and PCR cycles were performed according to the manufacturer’s instructions. Relative gene expression was analyzed using ΔΔC_t_ based fold-change calculations.

### Real time quantitative PCR analysis

Real-Time quantitative PCR (qPCR) was performed using LightCycler 480 and LightCycler 480 SYBR Green I Master reaction mixture (Roche, Penzberg, Germany). RNA was isolated as described above, whereupon 2 μg were used for cDNA-synthesis (SuperScript II Reverse Transcriptase, Invitrogen- Life technologies, Darmstadt, Germany).

Primer sequences are listed in Additional file 2: Table S2. cDNA was used at 1:10 dilution; targets with low gene expression were measured using 1:5 diluted cDNA. A cutoff for no detectable mRNA expression was set to a C_t_ level of 35 for further relative gene expression analysis (carried out as described before [18]), with ACTB, GAPDH and β2M as reference genes, according to MIQE guidelines [39].

### Protein extraction and quantification

Preparation of protein lysates was performed as described before [38]. Total protein content of cell lysates was estimated using bicinchoninic acid assay (BCA Kit, Sigma Aldrich, Taufkirchen, Germany).

### HMG CoA enzyme activity assay

Enzyme activity assay for HMG CoA reductase was determined for each biological replicate. Procedures were carried out as described before [38], using 100 μM of HMG CoA as initial substrate. Detection of MVL levels was monitored by an ultra-performance liquid chromatography (UPLC) system (Waters Acquity UPLC) using a 2.1 mm × 150 mm HSS PFP UPLC/MS cartridge (Waters, 1.8 μm ACQUITY UPLC HSS PFP Column) conducted at 45°C, which was directly coupled to a Quattro LC tandem mass spectrometer equipped with Z Spray ion source (Waters Xevo™ TQ-S).

### Quantitation of cellular proprotein convertase subtilisin/kexin type 9 (PCSK9)

Human PCSK9 in cell lysates of dermal fibroblasts was measured using Human PCSK9 ELISA Kit (Cell Biolabs, Inc., San Diego, USA) according to the manufacturer’s instructions. Protein lysates were obtained as described above. 5 μg of total cellular protein were used for enzyme immunoassay, measuring fibroblast samples in duplicates.

### Apolipoprotein E (APOE) genotyping

Genotyping of apolipoprotein E was performed according to the previously published method by Wenham *et al.*[40]. Isolated DNA of patients and controls was screened for apolipoprotein E sequence variations at position c.334 (T > C) and c.472 (C > T) by PCR on a LightCycler 480 (Roche, Applied Science). PCR was performed with fluorescence resonance energy transfer (FRET)-probes (fluorescein, LCRed 640). APOE genotypes were identified using melting curve analysis. Specific melting peak detection allows the characterization of homozygous or heterozygous sequence types, regarding melting temperatures and number of peaks.

### Statistical analysis

Experimental data are indicated as means ± S.E. Graphic data processing and statistics were performed with GraphPad Prism 5 (GraphPad Software, Inc., La Jolla, USA) using Mann-Whitney *U* Test (significance level p < 0.05).

## Results

### RT^2^ profiler PCR array

Analysis of 84 genes involved in cholesterol metabolism and lipoprotein signaling revealed altered mRNA levels between PXE fibroblasts and healthy controls grown in serum-free media for 24 h. Table 1 summarizes gene expressions regulated > 2-fold, or < 0.5-fold between PXE and control cells, as well as for siRNA-transfected cells. All results of PCR profiler array are provided in Additional file 3: Table S3. Fold-changes were characterized by comments (OKAY/A/B/C) based on the gene’s average threshold cycle as described in Table 1. Relative mRNA expression of proprotein convertase subtilisin/kexin type 9 (PCSK9) exhibited a 46-fold increase in PXE fibroblasts (p < 0.04) compared to healthy controls, whereas no differences were found between siRNA-transfected cells. Furthermore, 2.4-fold elevation of transmembrane 7 superfamily member 2 (TM7SF2) mRNA level in PXE cells revealed statistical significance (p < 0.04), which was also confirmed by trend with siRNA transfection (siABCC6/ siNK ratio: 2.1-fold). All other regulations were elevated or decreased by trend and no statistical significance was found due to insufficient sample size or low expression levels (see also comment description, Table 1. Array analysis showed distinct differences in apolipoprotein mRNA expressions between patient and control samples. A 124.6-fold increase was found for apolipoprotein L1 (APOL1) expression in PXE fibroblasts compared to control cells. Although, transcript levels of apolipoprotein D (APOD) were 2.3-fold higher in PXE fibroblasts. Expressions of apolipoprotein E (APOE), F (APOF) and L5 (APOL5) were decreased in PXE samples (patient/control ratio: 0.07; patient/control ratio: 0.5; patient/control ratio: 0.2, respectively), whereas a reduction in APOE expression was also confirmed by siRNA treatments (siABCC6/siNK ratio: 0.5).

**Table 1:** **RT**^**2 **^**profiler PCR array, lipoprotein signaling and cholesterol metabolism**

**Gene**	**Description**	**PXE/control**			**siABCC6/siNK**		
		**Fold change**^**a**^	**p-value ( *t *-test)**^**b**^	**Comments**^**c**^	**Fold change**^**a**^	**p-value ( *t *-test)**^**b**^	**Comments**^**c**^
ABCG1	ATP-binding cassette, sub-family G member 1	1.89	0.334	B	0.34	0.421	B
ANGPTL3	Angiopoietin-like 3	3.09	0.249	A	1.24	0.294	A
APOD	Apolipoprotein D	2.29	0.328	OKAY	0.92	0.796	OKAY
APOE	Apolipoprotein E	0.07	0.313	A	0.51	0.847	A
APOF	Apolipoprotein F	0.47	0.353	B	1.90	0.343	B
APOL1	Apolipoprotein L, 1	124.57	0.095	A	1.03	0.919	B
APOL5	Apolipoprotein L, 5	0.23	0.123	B	1.05	0.660	C
CEL	Carboxyl ester lipase (bile salt-stimulated lipase)	2.68	0.341	A	2.84	0.331	A
CXCL16	Chemokine (C-X-C motif) ligand 16	0.49	0.192	B	1.09	0.869	OKAY
CYP39A1	Cytochrome P450, family 39, subfamily A, polypeptide 1	3.64	0.068	A	1.05	0.809	B
CYP46A1	Cytochrome P450, family 46, subfamily A, polypeptide 1	0.57	0.250	B	0.47	0.192	B
HDLBP	High density lipoprotein binding protein	6.99	0.751	OKAY	0.99	0.989	OKAY
LRP1B	Low density lipoprotein receptor-related protein 1B	5.18	0.092	B	0.43	0.265	B
NPC1L1	NPC1 (Niemann-Pick disease, type C1, gene) like 1	0.36	0.161	B	0.89	0.562	B
OLR1	Oxidized low density lipoprotein (lectin-like) receptor 1	0.29	0.120	B	2.34	0.581	B
PCSK9	Proprotein convertase subtilisin/kexin type 9	**46.23**	**0.033**	A	1.18	0.820	B
SORL1	Sortilin-related receptor, L(DLR class) A repeats containing	1.77	0.112	B	2.18	0.390	B
TM7SF2	Transmembrane 7 superfamily member 2	**2.40**	**0.034**	OKAY	2.05	0.079	OKAY
TRERF1	Transcriptional regulating factor 1	4.93	0.222	OKAY	3.98	0.209	OKAY

Regulated gene expression of human dermal fibroblasts of PXE patients and healthy controls (PXE/controls) and siRNA transfected cells (siABCC6/siNK) cultivated without FCS for 24 h.

^a^fold-Change (2^(−ΔΔC_t_)) is the normalized gene expression (2^(−ΔC_t_)) in the test sample divided the normalized gene expression (2^(−ΔC_t_)) in the control sample.

^b^p-values are calculated based on a Student’s *t*-test of the replicate 2^(−ΔC_t_) values for each gene in the control group and treatment groups, and p values less than 0.05 are printed in bold.

^c^comments

OKAY: This gene's average threshold cycle is below 30 in the control and test samples.

A: This gene’s average threshold cycle is relatively high (>30) in either the control or the test sample, and is reasonably low in the other sample (<30).

B: This gene’s average threshold cycle is relatively high (>30), meaning that its relative expression level is low, in both control and test samples, and the p-value for the fold-change is either unavailable or relatively high (p > 0.05).

C: This gene’s average threshold cycle is either not determined or greater than the defined cut-off value (default 35), in both samples meaning that its expression was undetected, making this fold-change result erroneous and un-interpretable.

### ABCC6 gene expression in human dermal fibroblasts is highly increased under serum starvation

**Figure 1:**
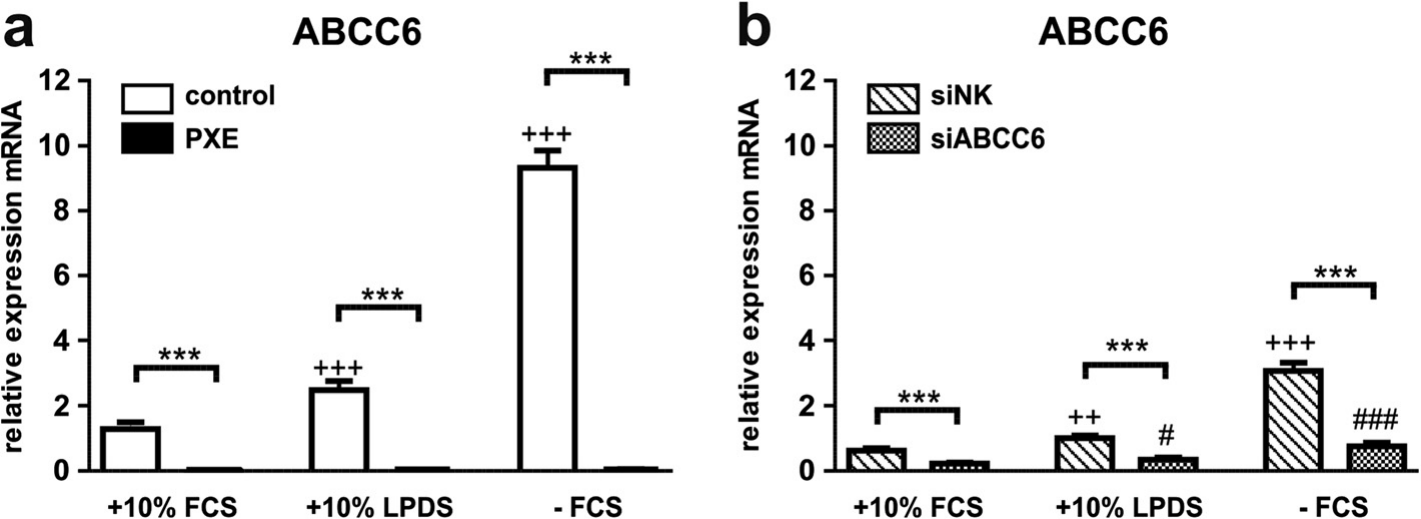
**Gene expression analysis of ABCC6. (a)** Quantification of ABCC6 mRNA expression in human dermal fibroblasts from healthy controls (n = 4; white) and PXE patients (n = 4; black) grown for 24 h under different cell culture conditions (+10% FCS, +10% LPDS, −FCS). **(b)** Effect of siRNA-mediated knockdown on ABCC6 gene expression: fibroblasts transfected with a scramble siRNA-negative control (siNK, n = 4; white-striped); ABCC6-specific siRNA-treated cells (siABCC6, n = 4; black-shaded) grown for 24 h under different cell culture conditions (+10% FCS, +10% LPDS, −FCS). Expression levels are normalized to reference gene expressions (ACTB, GAPDH, β2M). Data are presented in arbitrary units as means with corresponding standard error. Control: PXE ratio/siABCC6: siNK ratio: ***p < 0.0001; Control 10% FCS: 10% LPDS, −FCS ratio/siNK 10% FCS: 10% LPDS, −FCS ratio: +++p < 0.0001; ++p < 0.003; PXE 10% FCS: 10% LPDS, −FCS ratio/siABCC6 10% FCS: 10% LPDS, −FCS ratio: ###p < 0.0001; #p < 0.04.

As shown in Figure 1a, no mRNA expression of ABCC6 was quantifiable in PXE fibroblasts. Relative gene expression of ABCC6 was significantly increased in control fibroblasts under lipoprotein-deficient conditions (1.9-fold) and serum starvation (7.1-fold). A similar expression pattern was observed in siRNA-transfected control cells (Figure 1b; +10% LPDS: 1.6-fold; -FCS: 5.0-fold), while transcript levels of ABCC6 were down-regulated by 64.5% (10% FCS), 65% (10% LDPS) and 75.2% (−FCS) in siABCC6-transfected cells compared to siNK (p < 0.0001).

### Elevated HMG CoA reductase (HMGCR) enzyme activity in PXE fibroblasts

**Figure 2:**
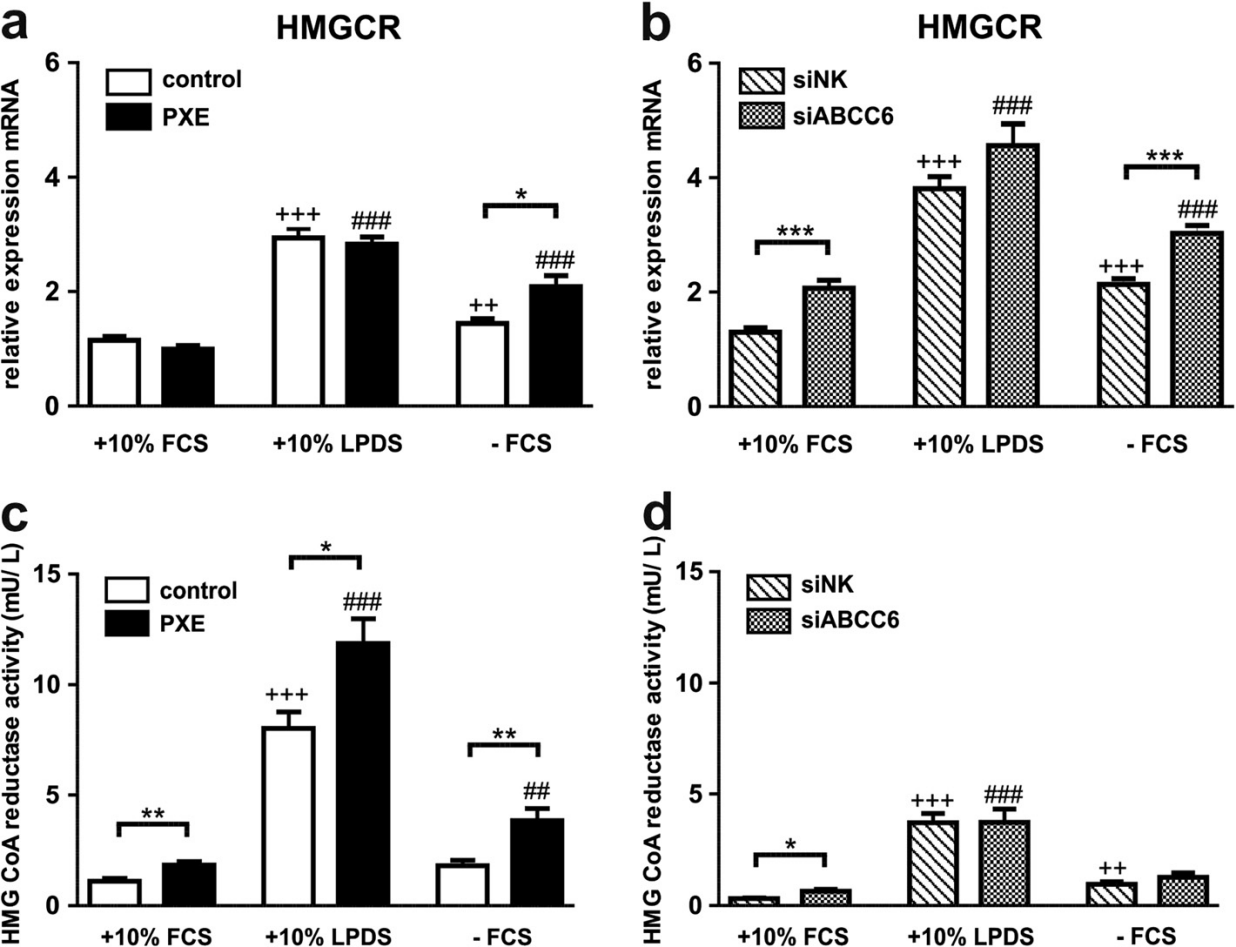
**Gene expression and enzyme activity analysis of HMG CoA reductase (HMGCR). (a)** Quantification of HMGCR mRNA expression in human dermal fibroblasts from healthy controls (n = 4; white) and PXE patients (n = 4; black) grown for 24 h under different cell culture conditions (+10% FCS, +10% LPDS, −FCS). **(b)** Effect of siRNA-mediated knockdown on ABCC6 gene expression: fibroblasts transfected with a scramble siRNA-negative control (siNK, n = 4; white-striped); ABCC6-specific siRNA-treated cells (siABCC6, n = 4; black-shaded) grown for 24 h under different cell culture conditions (+10% FCS, +10% LPDS, −FCS). Expression levels are normalized to reference gene expressions (ACTB, GAPDH, β2M). Data are presented in arbitrary units as means with corresponding standard error. Control: PXE ratio/siABCC6: siNK ratio: ***p < 0.0001; *p < 0.03; Control 10% FCS: 10% LPDS, −FCS ratio/siNK 10% FCS: 10% LPDS, −FCS ratio: +++p < 0.0001; ++p < 0.004; PXE 10% FCS: 10% LPDS, −FCS ratio/siABCC6 10% FCS: 10% LPDS, -FCS ratio: ###p < 0.0001 **(c, d)** Enzyme activity of HMG CoA reductase in fibroblasts of healthy controls, PXE patients **(c)** and siRNA- treated cells **(d)** measured by UPLC-MS/MS. Data are presented in arbitrary units as means with corresponding standard error. Control: PXE ratio/siABCC6: siNK ratio: **p < 0.003; *p < 0.03; Control 10% FCS: 10% LPDS, -FCS ratio/siNK 10% FCS: 10% LPDS, −FCS ratio: +++p < 0.0001; ++p < 0.002; PXE 10% FCS: 10% LPDS, −FCS ratio/siABCC6 10% FCS: 10% LPDS, −FCS ratio: ###p < 0.0001; ##p < 0.009.

Determination of HMGCR gene expression by qPCR revealed a significant increase in accordance with modified cell culture conditions (Figure 2a) in PXE and control fibroblasts compared to fibroblasts cultivated in 10% FCS (+10% LDPS: control, 2.6-fold; PXE, 2.9-fold; −FCS: control, 1.4-fold; PXE, 2.1-fold). Delipidation of fetal calf serum (LPDS) reduced free cholesterol by about 85% and LDL levels by about 96%, whereas triglyceride levels remained unchanged (data not shown).Measurements of HMG CoA reductase activity showed significantly elevated enzyme activities in PXE fibroblasts compared to control cells for each cell culture setting (Figure 2c; +10% FCS: 1.6-fold; +10% LDPS: 1.5-fold; −FCS: 2.1-fold). Activity was increased in LDPS or serum-free media in PXE fibroblasts compared to 10% FCS (+10% LDPS: PXE, 6.4-fold; −FCS: PXE, 2.1-fold) and a 7.1-fold induction was also observed in control fibroblasts grown in LPDS. However, overall enzyme activity was lower in siRNA-transfected samples compared to PXE and control fibroblasts (Figure 2b). Here, increasing enzyme activities were also detected in LPDS (siNK, 11.6-fold; siABCC6, 5.7-fold) and serum-free treatment of siRNA control (4.0-fold) compared to 10% FCS. However, no alterations were found between siNK and siABCC6 samples, except for cells cultivated in 10% FCS (siABCC6/ siNK ratio: 2.0).

### Lipoprotein deficiency and serum starvation induce cholesterol biosynthetic gene expression in human dermal fibroblasts

**Figure 3:**
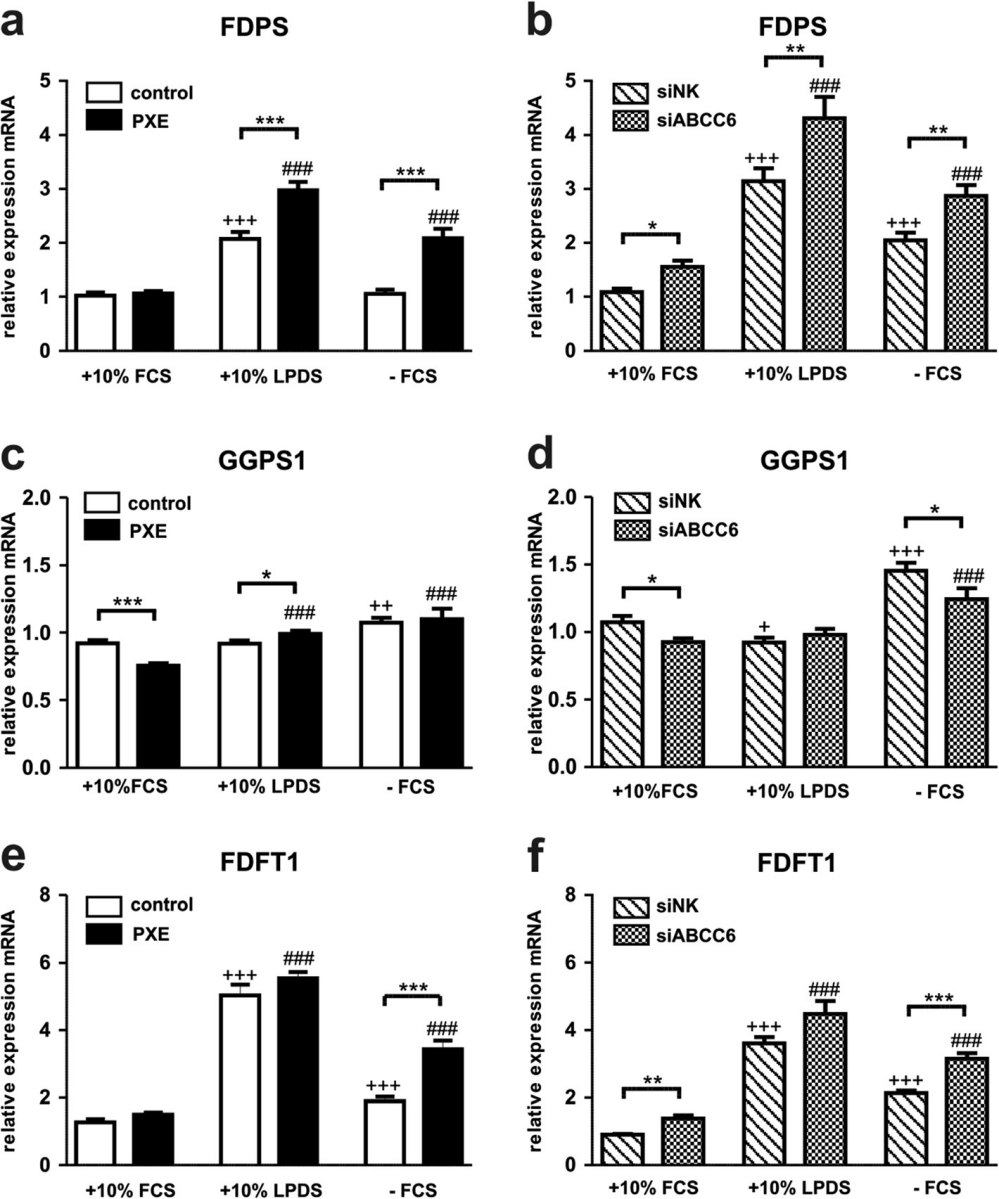
**Gene expression analysis of cholesterol biosynthesis I: FDPS, GGPS1 and FDFT1.** Quantification of **(a, b)** Farnesyl diphosphate synthase (FDPS), **(c, d)** Geranylgeranyl diphosphate synthase (GGPS1) and **(e, f)** Squalene synthase (FDFT1) mRNA expression in human dermal fibroblasts from healthy controls (n = 4; white), PXE patients (n = 4; black), scramble siRNA-negative control (siNK, n = 4; white-striped) and ABCC6-specific siRNA-treated cells (siABCC6, n = 4; black-shaded) grown for 24 h under different cell culture conditions (+10% FCS, +10% LPDS, −FCS). Expression levels are normalized to reference gene expressions (ACTB, GAPDH, β2M). Data are presented in arbitrary units as means with corresponding standard error. Control: PXE ratio/siABCC6: siNK ratio: ***p < 0.0003; **p < 0.003; *p < 0.04; Control 10% FCS: 10% LPDS, −FCS ratio/siNK 10% FCS: 10% LPDS, −FCS ratio: +++p < 0.0006; ++p < 0.003; +p < 0.05; PXE 10% FCS: 10% LPDS, −FCS ratio/siABCC6 10% FCS: 10% LPDS, -FCS ratio: ###p < 0.0007.

**Figure 4:**
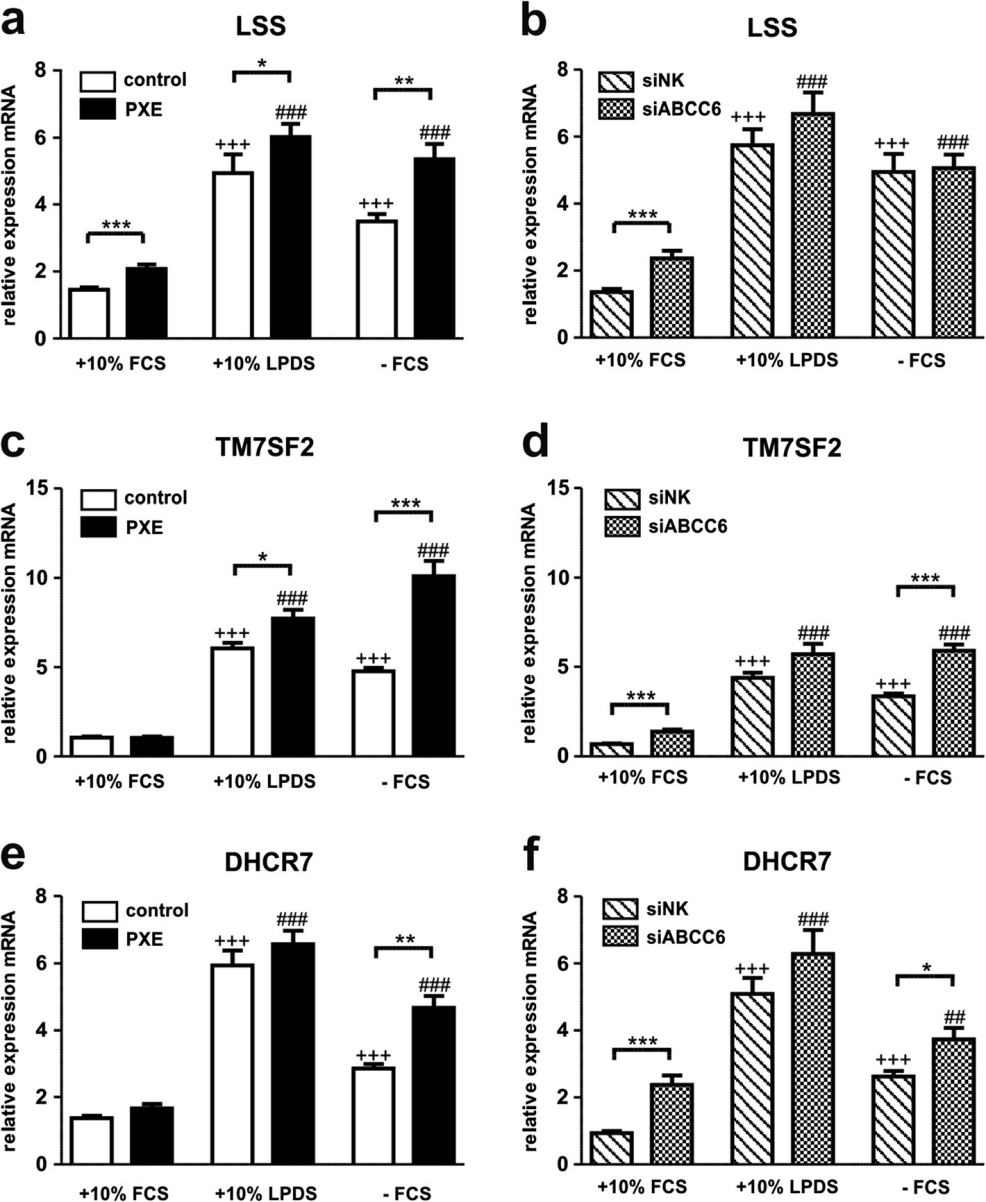
**Gene expression analysis of cholesterol biosynthesis II: LSS, TM7SF2 and DHCR7.** Quantification of **(a, b)** Lanosterol synthase (LSS), **(c, d)** transmembrane 7 superfamily member 2 (TM7SF2) and **(e, f)** 7-dehydrocholesterol reductase (DHCR7) mRNA expression in human dermal fibroblasts from healthy controls (n = 4; white), PXE patients (n = 4; black), scramble siRNA-negative control (siNK, n = 4; white-striped) and ABCC6-specific siRNA-treated cells (siABCC6, n = 4; black-shaded) grown for 24 h under different cell culture conditions (+10% FCS, +10% LPDS, −FCS). Expression levels are normalized to reference gene expressions (ACTB, GAPDH, β2M). Data are presented in arbitrary units as means with corresponding standard error. Control: PXE ratio/siABCC6: siNK ratio: ***p < 0.0005; **p < 0.005; *p < 0.03; Control 10% FCS: 10% LPDS, −FCS ratio/siNK 10% FCS: 10% LPDS, −FCS ratio: +++p < 0.0001; PXE 10% FCS: 10% LPDS, −FCS ratio/siABCC6 10% FCS: 10% LPDS, -FCS ratio: ###p < 0.0001; ##p < 0.002.

In addition to HMGCR, marking the rate-limiting step in cholesterol biosynthesis, gene expression of farnesyl diphosphate synthase (FDPS), geranylgeranyl diphosphate synthase 1 (GGPS1), squalene synthase (FDFT1), lanosterol synthase (LSS), transmembrane 7 superfamily member 2 (TM7SF2) and 7-dehydrocholesterol reductase (DHCR7) were analyzed (Figures 3 and 4).Under serum-free conditions, PXE fibroblasts exhibited a significant increase in all targets mentioned above (except GGPS1) compared to healthy controls, which were mostly confirmed by ABCC6 knockdown (Figures 3 and 4; FDPS: PXE, 2.0-fold; siABCC6, 1.4-fold; FDFT1: PXE, 1.8-fold; siABCC6: 1.5-fold; LSS: PXE, 1.5-fold; siABCC6: 1.5-fold; TM7SF2: PXE, 2.1-fold; siABCC6, 1.8-fold; DHCR7: PXE, 1.6-fold; siABCC6, 1.4-fold; p values are given in Figures 3 and 4).Using LPDS, transcript levels of the analyzed targets in PXE cells were slightly elevated or unchanged relative to healthy controls (Figures 3 and 4; FDPS: PXE, 1.4-fold; FDFT1: PXE, 1.1-fold; LSS: PXE, 1.2-fold; TM7SF2: PXE, 1.3-fold; DHCR7: PXE, 1.1-fold; p-values are given in Figure 3.18, 3.19). ABCC6-silenced fibroblasts showed even slightly increased or unaltered mRNA levels under LPDS cultivation in comparison to siRNA controls (Figures 3 and 4).Cell cultivation in 10% LPDS increased mRNA expression of all targets in PXE and control fibroblasts, as well as in siRNA treated cells in comparison to 10% FCS (Figures 3 and 4). Only a slight increase under serum-free culture conditions was found for gene expression analysis of GGPS1 in patients and controls (Figure 3c, d).

Additionally, transcript levels (except FDPS mRNA expression of control fibroblasts) increased significantly under serum deprivation (-FCS) compared to standard cultivation in 10% FCS.

### Sterol regulatory element-binding protein 2 (SREBP2) and sterol regulatory element-binding factor 1 (SREBF1) increased in response to lipoprotein reduction and serum withdrawal

**Figure 5:**
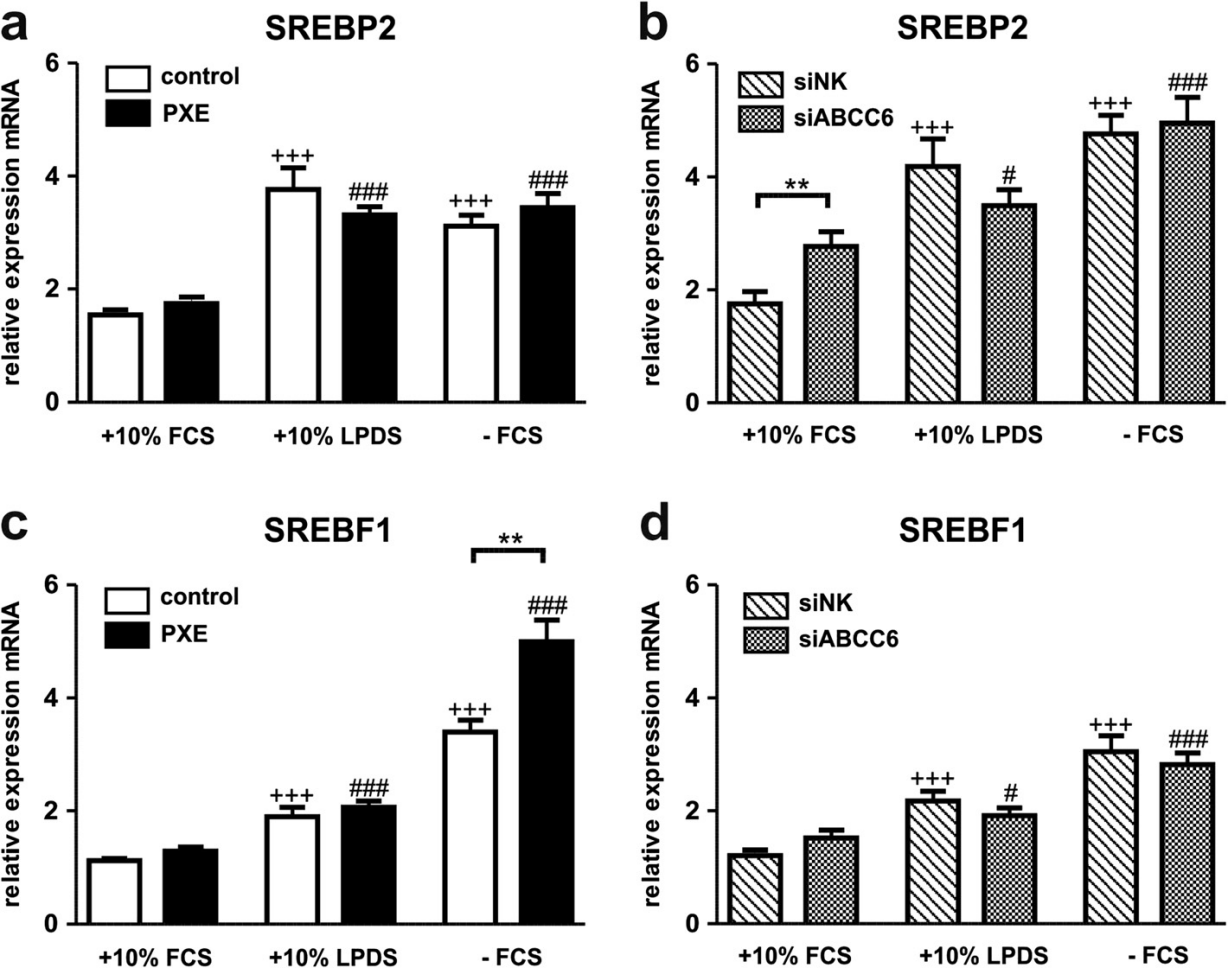
**Gene expression analysis of Sterol regulatory element-binding protein 2 (SREBP2) and sterol regulatory element binding factor 1 (SREBF1).** Quantification of **(a, b)** Sterol regulatory element-binding protein 2 (SREBP2) and **(c, d)** sterol regulatory element binding factor 1 (SREBF1) mRNA expression in human dermal fibroblasts from healthy controls (n = 4; white), PXE patients (n = 4; black), scramble siRNA-negative control (siNK, n = 4; white-striped) and ABCC6-specific siRNA-treated cells (siABCC6, n = 4; black-shaded) grown for 24 h under different cell culture conditions (+10% FCS, +10% LPDS, −FCS). Expression levels are normalized to reference gene expressions (ACTB, GAPDH, β2M). Data are presented in arbitrary units as means with corresponding standard error. Control: PXE ratio/siABCC6: siNK ratio: **p < 0.006; Control 10% FCS: 10% LPDS, −FCS ratio/siNK 10% FCS: 10% LPDS, −FCS ratio: +++p < 0.0002; PXE 10% FCS: 10% LPDS, −FCS ratio/siABCC6 10% FCS: 10% LPDS, -FCS ratio: ###p < 0.0008; #p < 0.03.

Sterol regulatory element-binding protein 2 (SREBP2) increases LDLR and PCSK9 gene expression, as a sterol sensitive regulator in cholesterol homeostasis [35]. Induction of SREBP2 and SREBF1 mRNA expression was observed in 10% LPDS and serum-free media compared to 10% FCS (Figure 5a-d). No significant changes of SREBP2 transcript levels were detected between PXE and control cells (Figure 5a). However, SREBF1 mRNA was significantly elevated in PXE fibroblasts under serum starvation (patient/control ratio: 1.5).

### Altered gene and protein expression of proprotein convertase subtilisin/kexin type 9 (PCSK9) and low density lipoprotein receptor (LDLR) in PXE fibroblasts

**Figure 6:**
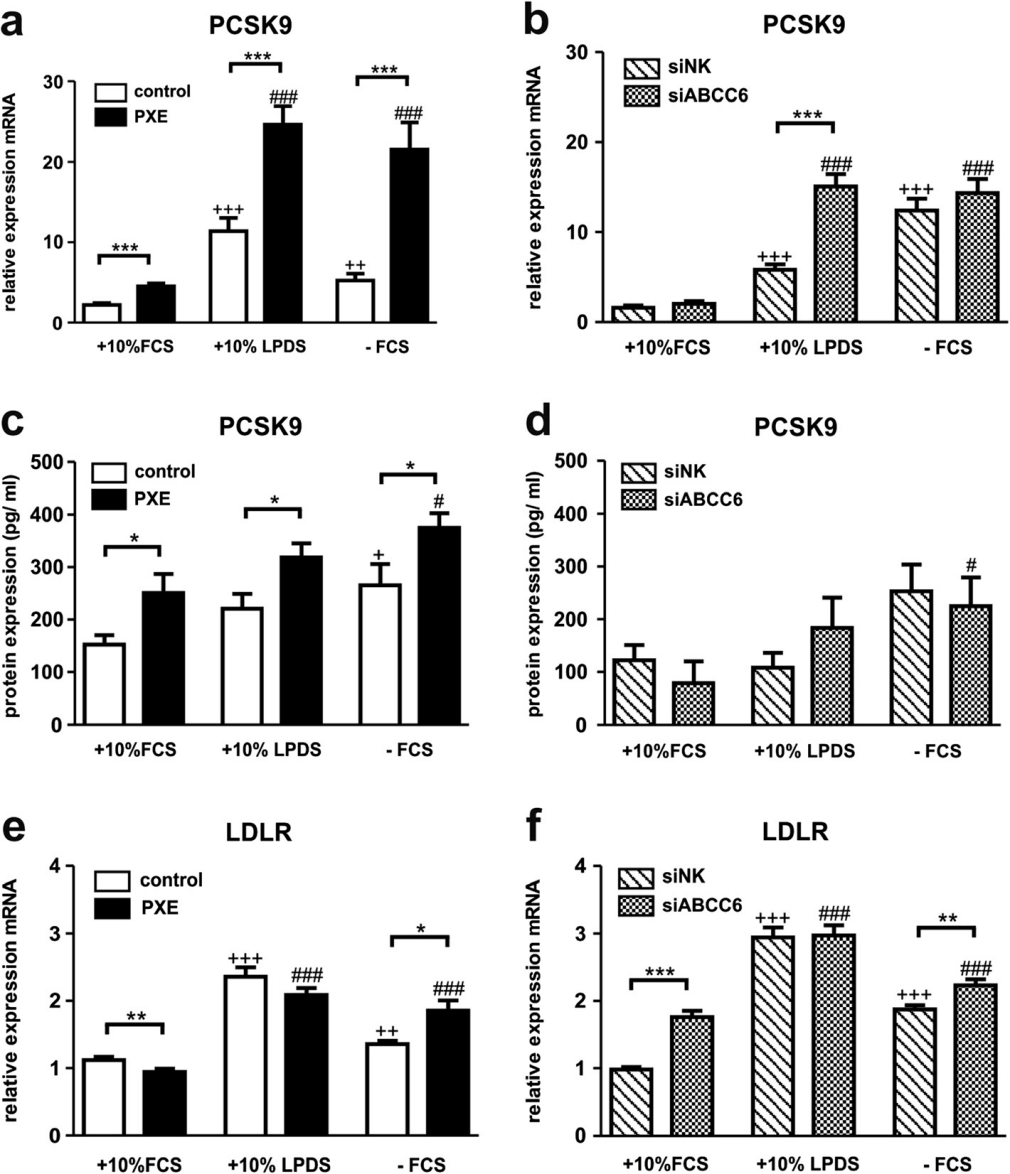
**Gene and protein expression analysis of proprotein convertase subtilisin/kexin type 9 (PCSK9) and low density lipoprotein receptor (LDLR).** Quantification of **(a, b)** mRNA **(c, d)** PCSK9 protein content (pg/ ml) and **(e, f)** LDLR mRNA expression in human dermal fibroblasts from healthy controls (n = 4; white), PXE patients (n = 4; black), scramble siRNA-negative control (siNK, n = 4; white-striped) and ABCC6-specific siRNA-treated cells (siABCC6, n = 4; black-shaded) grown for 24 h under different cell culture conditions (+10% FCS, +10% LPDS, −FCS). Gene expression levels are normalized to reference gene expressions (ACTB, GAPDH, ß2M). Cell lysates for ELISA analysis were pooled from three biological replicates, measured in duplicates. Data are presented in arbitrary units as means with corresponding standard error. Control: PXE ratio/siABCC6: siNK ratio: ***p < 0.0001; **p < 0.003; *p < 0.05; Control 10% FCS: 10% LPDS, −FCS ratio/siNK 10% FCS: 10% LPDS, −FCS ratio: +++p < 0.0001; ++p < 0.002; +p < 0.02; PXE 10% FCS: 10% LPDS, -FCS ratio/siABCC6 10% FCS: 10% LPDS, -FCS ratio: ###p < 0.0001; #p < 0.03.

Gene expression analysis of PCSK9 by RT^2^ Profiler PCR Array revealed a 46-fold increase in PXE fibroblasts compared to healthy controls grown under serum-free conditions (see section RT^2^ Profiler PCR Array).Verification by further qPCR showed significantly elevated mRNA expression in PXE fibroblasts for all cell culture settings in comparison to controls (Figure 6a: +10% FCS: 2.0-fold; +10% LDPS: 2.2-fold; −FCS: 4.1-fold). Moreover, PCSK9 expression was highly induced under lipoprotein-deficient and serum-free conditions in controls and PXE fibroblasts compared to 10% FCS (10% LPDS: control, 5.1-fold; PXE, 5.5-fold; −FCS: control, 2.4-fold; PXE, 4.8-fold). Serum-dependent induction of PCSK9 expression was also confirmed in siRNA-transfected fibroblasts (Figure 6b), whereas transcript levels of ABCC6-silenced cells (grown in 10% LPDS) were 2.6-fold higher than negative controls. Protein expression of PCSK9 was significantly increased in PXE fibroblasts compared to controls in every cell culture setting (Figure 6c: +10% FCS: 1.7-fold; +10% LDPS: 1.6-fold; −FCS: 1.4-fold). Protein levels in siRNA treated cells were increased by trend under LDPS and without FCS. However, no significant changes were found between treatments, except for a slight increase in LPDS according to mRNA expression.

PCSK9 plays a pivotal role in lipoprotein regulation promoting LDLR degradation [41]. Measurements displayed increasing mRNA levels of LDLR under lipoprotein deficiency and serum starvation in PXE, control and siRNA-treated fibroblasts (Figure 6e, f). Moreover, gene expression was enhanced in PXE and ABCC6-silenced cells grown in serum-free media compared to controls.

### Variations in apolipoprotein gene expression profiles between PXE fibroblasts and healthy controls

**Figure 7:**
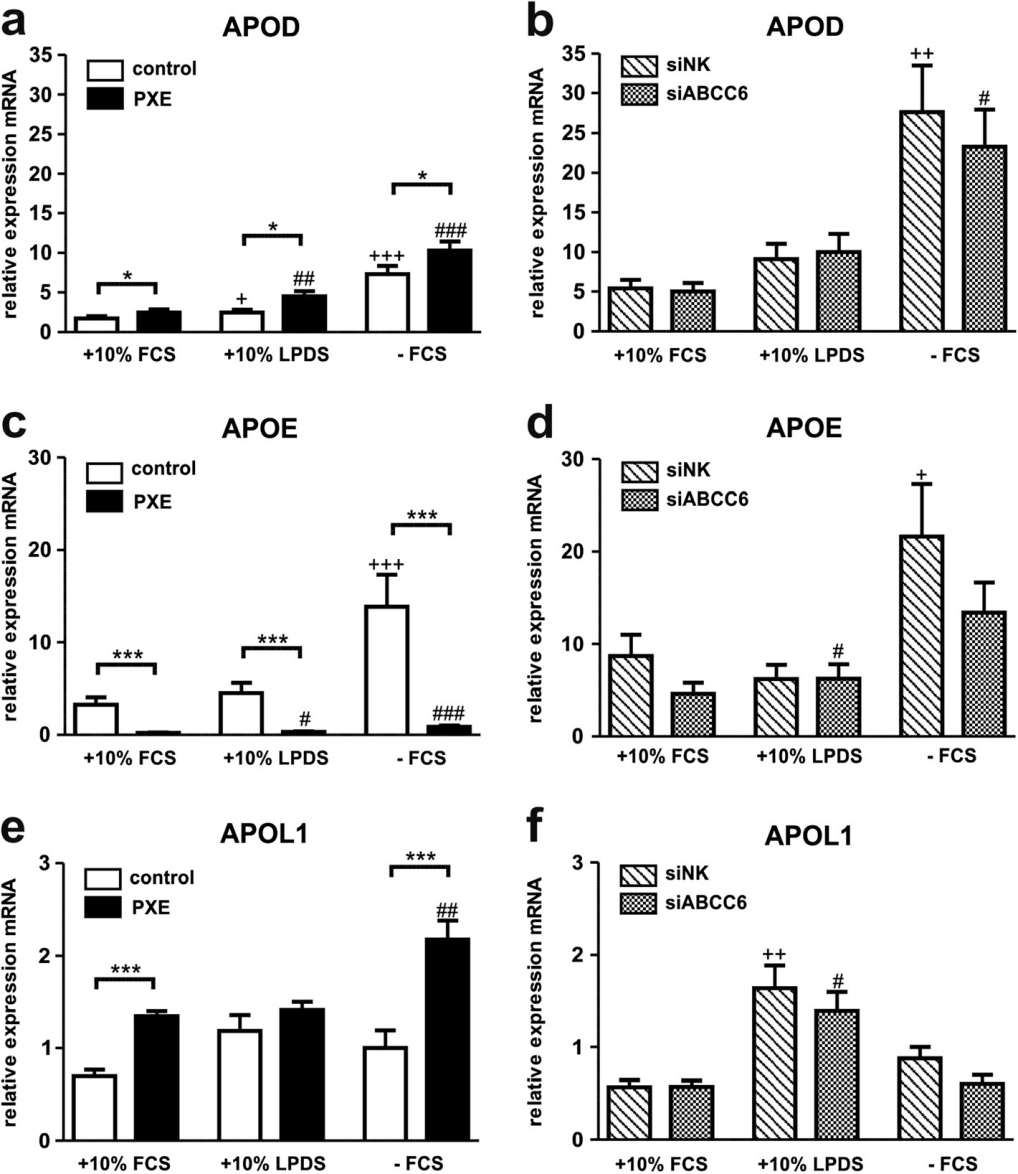
**Gene expression analysis of Apolipoproteins: APOD, APOE and APOL1.** Quantification of **(a, b)** apolipoprotein D (APOD), **(c, d)** apolipoprotein E (APOE) and **(e, f)** apolipoprotein L1 (APOL1) mRNA expression in human dermal fibroblasts from healthy controls (n = 4; white), PXE patients (n = 4; black), scramble siRNA-negative control (siNK, n = 4; white-striped) and ABCC6-specific siRNA-treated cells (siABCC6, n = 4; black-shaded) grown for 24 h under different cell culture conditions (+10% FCS, +10% LPDS, −FCS). Expression levels are normalized to reference gene expressions (ACTB, GAPDH, β2M). Data are presented in arbitrary units as means with corresponding standard error. Control: PXE ratio/siABCC6: siNK ratio: ***p < 0.0001; *p < 0.03; Control 10% FCS: 10% LPDS, -FCS ratio/siNK 10% FCS: 10% LPDS, −FCS ratio: +++p < 0.0004; ++p < 0.003; +p < 0.04; PXE 10% FCS: 10% LPDS, −FCS ratio/siABCC6 10% FCS: 10% LPDS, -FCS ratio: ###p < 0.0001; ##p < 0.005; #p < 0.05.

Array analysis indicated differences in gene expression levels of APOD, APOE and APOL1 between patients’ fibroblasts and controls (see section RT^2^ Profiler PCR Array). As shown in Figure 7a, PXE fibroblasts exhibited increased APOD expression compared to controls, apparently independently of cell culture conditions. However, no differences were found between siRNA-transfected cells (Figure 5b), whereas mRNA levels were significantly elevated in serum-free media compared to 10% FCS (siNK, 5.1-fold, p < 0.005; siABCC6, 4.7-fold, p < 0.05).

Strongly depleted gene expression was observed for APOE in PXE fibroblasts (Table 1) by array analysis. This was confirmed by further qPCR which revealed an overall decrease of 93- 94% in APOE transcript levels in comparison to controls (Figure 7c). Though, no significant alterations were detected between ABCC6-silenced fibroblasts and negative controls, except slightly decreased expressions of APOE in 10% FCS and without serum supplementation (Figure 7d). In general, APOE gene expression was highly induced under serum starvation in control fibroblasts (4.3-fold) and siRNA negative controls (2.5-fold; Figure 7c, d). Measurements of APOE transcript levels were accompanied by genomic sequence analysis of APOE allele variations in patients and control samples (Additional file 4: Table S4). Two PXE patients and controls were found to carry the abundant ϵ3 isoform homozygously, whereas one PXE patient carried the ϵ3/ϵ2 alleles in heterozygous state. Two controls were found to bear the heterozygous ϵ3/ϵ4 genotype. Additionally, the ϵ2 isoform was detected for PXE patient 1 in homozygous state.

PXE fibroblasts showed increased APOL1 gene expression in serum-free media in comparison to controls (Table 1). These results were confirmed by further RTQ-PCR measurements, as shown in Figure 7e. APOL1 expression was significantly higher in patients’ cells compared to controls in 10% FCS (1.9-fold) and -FCS (2.2-fold), whereas cultivation in lipoprotein-deficient serum showed just a slight increase. However, no significant alterations were found between siRNA-treated fibroblasts (Figure 7f).

### Gene expression of CYP27A1- a marker for cholesterol hydroxylation and bile acid synthesis

**Figure 8:**
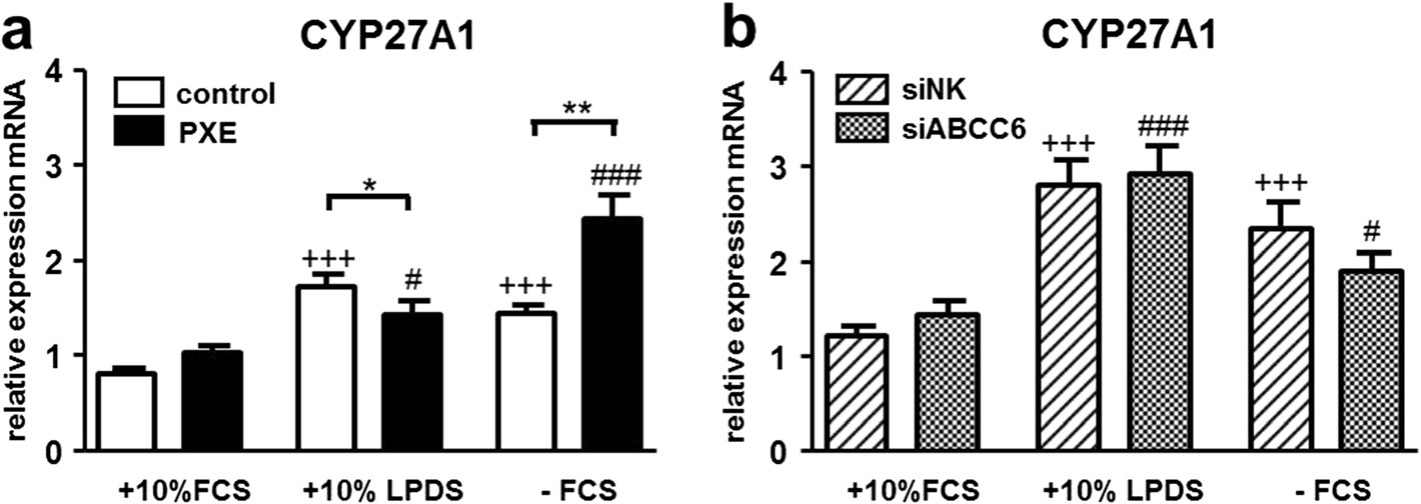
**Gene expression analysis of CYP27A1.** Quantification of **(a, b)** CYP27A1a mRNA expression in human dermal fibroblasts from healthy controls (n = 4; white), PXE patients (n = 4; black), scramble siRNA-negative control (siNK, n = 4; white-striped) and ABCC6-specific siRNA-treated cells (siABCC6, n = 4; black-shaded) grown for 24 h under different cell culture conditions (+10% FCS, +10% LPDS, −FCS). Expression levels are normalized to reference gene expressions (ACTB, GAPDH, β2M). Data are presented in arbitrary units as means with corresponding standard error. Control: PXE ratio/siABCC6: siNK ratio: **p < 0.002; *p < 0.05; Control 10% FCS: 10% LPDS, −FCS ratio/siNK 10% FCS: 10% LPDS, −FCS ratio: +++p < 0.0002; PXE 10% FCS: 10% LPDS, −FCS ratio/siABCC6 10% FCS: 10% LPDS, -FCS ratio: ###p < 0.0001; #p < 0.04.

Gene expression of cytochrome P450, family 27, subfamily A, polypeptide 1 (CYP27A1) was significantly induced in cells grown in 10% LPDS and without serum supplementation (Figure 8a, b). PXE fibroblasts were found to exhibit higher transcript levels under serum starvation in comparison to healthy controls (1.7-fold), whereas expression was slightly decreased in LPDS (20%; Figure 8a). However, no differences were detected between ABCC6-silenced fibroblasts and controls (Figure 8b).

### Targeting ABCC2 and ABCC3- important members of bile salt transport

**Figure 9:**
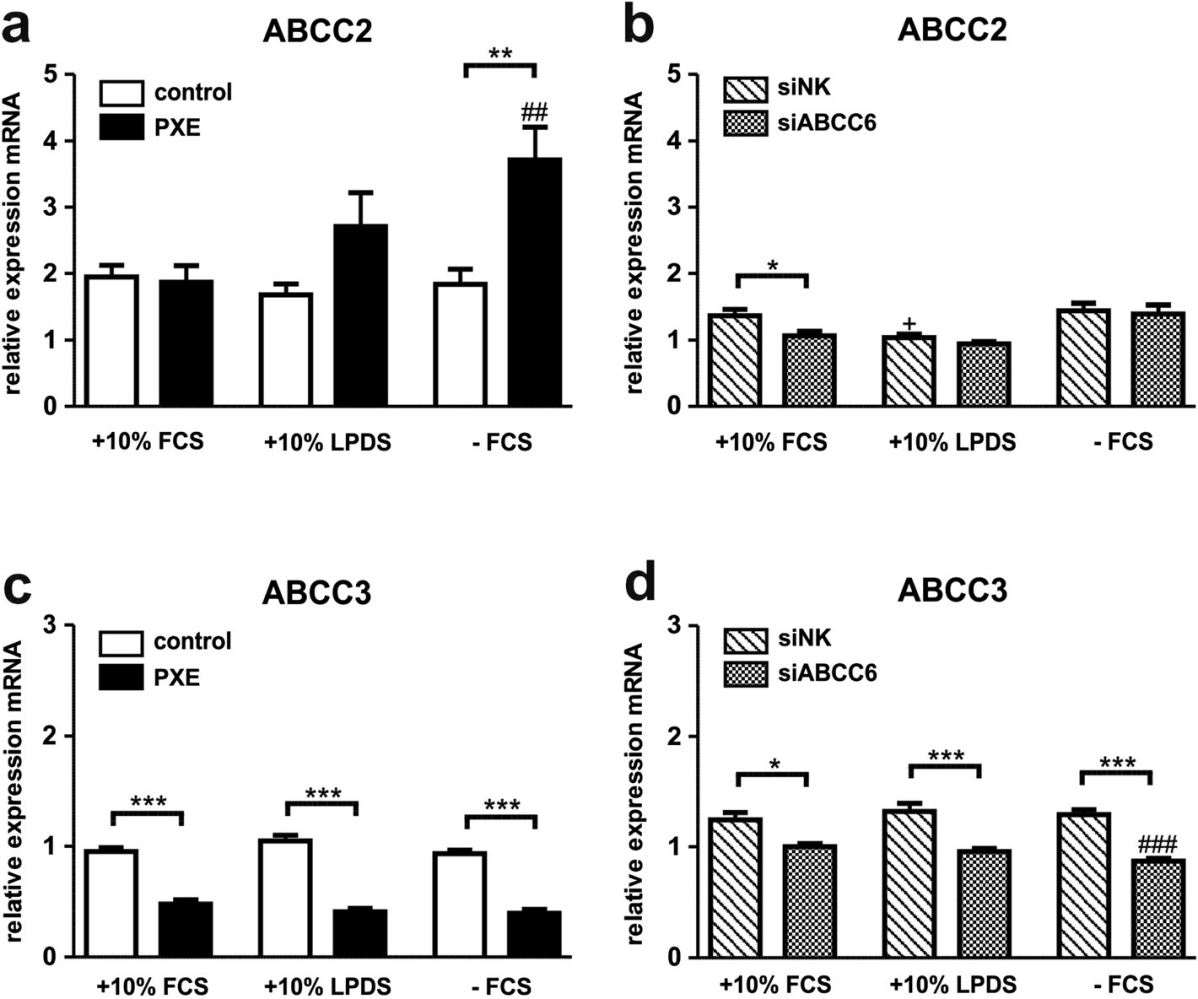
**Gene expression analysis of ABCC-Transporters ABCC2 and ABCC3.** Quantification of **(a, b)** ABCC2 and **(c, d)** ABCC3 mRNA expression in human dermal fibroblasts from healthy controls (n = 4; white), PXE patients (n = 4; black), scramble siRNA-negative control (siNK, n = 4; white-striped) and ABCC6-specific siRNA-treated cells (siABCC6, n = 4; black-shaded) grown for 24 h under different cell culture conditions (+10% FCS, +10% LPDS, −FCS). Expression levels are normalized to reference gene expressions (ACTB, GAPDH, β2M). Data are presented in arbitrary units as means with corresponding standard error. Control: PXE ratio/siABCC6: siNK ratio: ***p < 0.0002; **p < 0.005; *p < 0.02; Control 10% FCS: 10% LPDS, −FCS ratio/siNK 10% FCS: 10% LPDS, −FCS ratio: +p < 0.03; PXE 10% FCS: 10% LPDS, −FCS ratio/siABCC6 10% FCS: 10% LPDS, -FCS ratio: ###p < 0.0009; ##p < 0.005.

PXE fibroblasts grown in serum-free media showed 2.0-fold increase in ABCC2 gene expression, compared to 10% FCS and control cells (Figure 9a). On the other hand, the expression pattern of control fibroblasts and siRNA-transfected cells was unchanged or moderately depleted between cell culture settings (Figure 9a, b).Analysis of ABCC3 mRNA expression revealed significant reduction in PXE and ABCC6-silenced cells compared to controls (Figure 9c, d). A 50- 60% reduction was found in PXE fibroblasts, whereas ABCC6-knockdown exhibited 20- 35% depletion. These regulations were found to be independent of cell culture conditions.

## Discussion

In this study, we describe for the first time alterations in cellular cholesterol and lipoprotein metabolism in human dermal fibroblasts from patients with *Pseudoxanthoma elasticum*. Gene expression analysis of 84 targets involved in cholesterol biosynthesis and lipoprotein assembly indicated dysregulations in response to ABCC6 deficiency. Highly increased or downregulated genes were further examined under different cell culture conditions (+10% FCS, +10% LPDS, −FCS), inducing HMG CoA reductase activity. However, a few results from array analysis could not be confirmed by additional qPCR, pointing towards the need for additional data verification of microarray analysis by secondary methods [42].

Transcript levels of ABCC6 were strongly increased in lipoprotein-deficient serum (LPDS) and under serum-free conditions in control and siRNA treated cells. ABCC6 silencing reached 65- 75%, whereas mRNA expression levels of ABCC6 were overall diminished in siRNA treatments in comparison to controls without transfection. To what extent this was caused by liposome-based transfection, or enlarged cultivation time (+24 h) for siRNA treatments is not clear. As shown before, no ABCC6 mRNA could be detected in fibroblasts from PXE patients [18].

**Figure 10:**
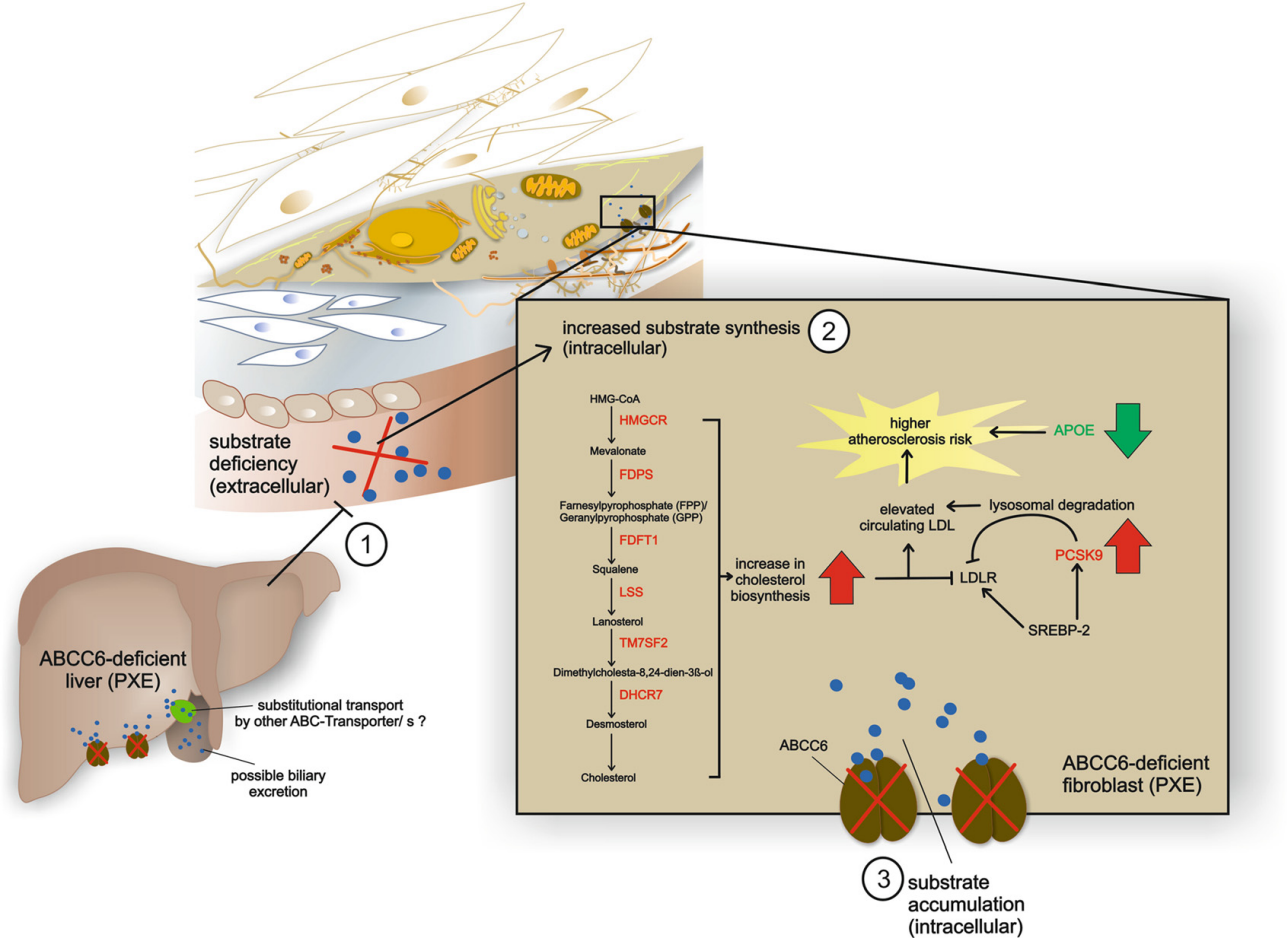
**Proposed molecular mechanisms underlying the dysregulations in cholesterol and lipoprotein metabolism in ABCC6-deficient PXE fibroblasts.** In case of ABCC6-deficiency in the liver, the potential substrate(s) of ABCC6 is/are missing in the circulation (1). Hepatocytes might possess substitutional routes to prevent possible toxic substrate accumulations (e.g. biliary excretion by other ABC transporter/s). Insufficient substrate supply by the liver then induces substrate biosynthesis in ABCC6-deficient dermal fibroblasts (e.g. cholesterol biosynthesis) (2). Due to a functional loss of ABCC6, newly synthesized metabolites accumulate intracellularly (3) and might provide an additional trigger for dysregulations in cholesterol homeostasis in addition to insufficient hepatic substrate supply.

The main results of this study are summarized in Figure 10. We used human dermal fibroblasts as a cellular model for ABCC6 deficiency in peripheral cells, in addition to their functional role in extracellular matrix assembly. Previously published data showed that fibroblasts from PXE patients exhibit a characteristic phenotype distinct from healthy controls, affecting ABC transporter expression [18], calcification processes [43], or extracellular matrix organization [44,45]. ABCC6 is predominately expressed in the liver and kidney and transcript levels in fibroblasts were shown to be significantly lower [18]. The pathogenesis of PXE is currently explained by the “metabolic hypothesis” and the “peripheral cell hypothesis” [46,47]. In case of ABCC6 deficiency in the liver, potential substrate(s) of ABCC6 is/are missing in the circulation, which leads to the ectopic mineralization processes in peripheral tissues. However, the liver is not affected in PXE patients and hepatocytes might possess substitutional routes to prevent possible toxic substrate accumulations (e.g. biliary excretion by other ABC transporter/s). Theoretically, mineralization can occur due to substrate deficiency in peripheral tissues, which normally counteracts calcification processes. On the other hand, mineralization can be forced by intracellular substrate biosynthesis in ABCC6-deficient cells (e.g. dermal fibroblasts), which is induced by insufficient substrate supply by the liver and subsequent harmful accumulation affecting intracellular pathways. Recent studies have demonstrated that cholesterol and its precursors are mainly generated in the liver (supplying extrahepatic tissues) [48,49], but can even be newly synthesized by peripheral cells, e.g. by human dermal fibroblasts [50,51].

Many ABC transporters are involved in lipid homeostasis, including RCT and phospholipid or cholesterol efflux [3,26]. Recent studies showed variations in plasma and serum lipoprotein and triglyceride concentrations in PXE patients [27,29]. We investigated human dermal fibroblasts from PXE patients and healthy controls under standard cell culture conditions and by inducing cholesterol biosynthesis (HMGCR activity) through lipoprotein deficiency (LPDS) or serum-free (−FCS) cultivation, as shown before [36,50]. Measurement of HMG CoA reductase activity showed for the first time a significant increase in cholesterol biosynthesis rates in PXE fibroblasts in comparison to healthy controls, which was observed under all tested cell culture settings. These data were confirmed by further qPCR measurements of important targets in cholesterol biosynthesis (FDPS, GGPS1, FDFT1, LSS, TM7SF2, DHCR7), which revealed overall increased transcript levels in PXE patients, predominately under serum starvation and using LPDS. Therefore, our data indicate a conceivable role for ABCC6 in human cellular cholesterol and lipoprotein metabolism, observing significant differences between PXE fibroblasts and healthy controls. The results of our study provide a first hint, uncovering the molecular mechanisms underlying the positive outcome of statin treatment observed in Abcc6^−/−^ mice [31,52]. Moreover, newly published data including whole-genome sequencing of 89 individuals of polar and brown bears revealed that ABCC6 is one of the important genes which has been under high positive selection in polar bears, enabling to deal with life-long elevated LDL levels that are associated with high risk of heart disease in humans [53].

Most of these results were approved by siRNA knockdown experiments. However, highest ABCC6 mRNA expression under serum starvation could not directly be correlated to the highest HMGCR activity, which was found in 10% LPDS. Nevertheless, higher cholesterol biosynthesis in PXE could be an important pro-atherosclerotic factor also affecting calcification progress. The missing correlation of HMG CoA activity and ABCC6 mRNA levels could be explained by further serum factors which may participate in transcription control or act as direct substrate/s for ABCC6. Therefore, cell cultivation in LPDS and without FCS could underlie different regulations, while substrate/s of ABCC6 could be still existent in LPDS.

Gene expression array data indicated strongly increased transcript levels for the proprotein convertase subtilisin/kexin type 9 (PCSK9) in PXE fibroblasts, which was also confirmed by additional qPCR and ELISA measurements. Gene expression levels of the LDLR were slightly elevated in PXE fibroblasts and ABCC6-silenced cells compared to controls.

Our study is the first linking ABCC6 deficiency to increased PCSK9 mRNA and protein levels. Pisciotta *et al.* examined a hypercholesterolemic PXE patient, who was compound-heterozygous for two ABCC6 mutations (p.S317R and p.R1141X) and for further mutations in candidate genes causing autosomal co-dominant hypercholesterolemia [54]. A heterozygous LDLR mutation (p.R574H) was found in this patient, whereas no sequence variations were identified in the PCSK9 gene. PCSK9 is mainly expressed in the liver, but was also found in human fibroblasts [55] (similar to the expression pattern of ABCC6) and plays an important role in LDL receptor lysosomal degradation [41]. Modification of serum lipoprotein content, as well as serum withdrawal, induced PCSK9 gene and protein expression in our experiments. PCSK9 was shown to be increased by peroxisome proliferator-activated receptor gamma (PPARγ) ligands [56], bisphosphonate administration [57], statin use or by LXR-agonists [58,59]. On the other hand, farnesoid X Receptor (FXR) agonists, 24(S), 25-epoxycholesterol and the lanosterol synthase inhibitor OSCi decreased PCSK9 expression in human hepatocytes and vascular smooth muscle cells, respectively [58,59]. Interestingly, induction of PCSK9 expression by PPARγ ligands was supposed to be dependent on extracellular signal regulated kinases 1 and 2 (ERK 1/2) inhibition [56]. The ERK1/2- hepatocyte nuclear factor 4 alpha (HNF4α)-axis has been previously described as an important factor for ABCC6 transcription control and might be an additional link to the observed elevated PCSK9 levels in PXE fibroblasts [60]. In addition to increased cholesterol biosynthesis found in PXE fibroblasts, elevated PCSK9 levels could force atherogenesis and cardiovascular risk in PXE patients. Thus, known regulators for PCSK9 (e.g. oxysterols, bile acids) should be investigated as potential substrate/s for ABCC6 in future studies.

Decreased levels of apolipoprotein E (APOE) are an additional risk factor for the development of atherosclerosis. APOE plays an important role in chylomicron and very low density lipoprotein (VLDL) recycling, interacting with LDL receptors in the liver [61]. Single nucleotide variants in APOE were described as determinants for receptor interaction rates and risk factors for atherosclerosis, hypercholesterolemia or Alzheimer disease [62]. We found different APOE isoforms, like heterozygous ϵ3/ϵ2 and homozygous ϵ2 alleles in PXE patients and heterozygous ϵ3/ϵ4 in controls, in addition to the abundant homozygous ϵ3 isoform. The presence of at least one ϵ2 allele was associated with higher APOE levels and a lower risk for CHD, whereas at least one allele of the isoform ϵ4 was shown to predict lower APOE levels and a higher potential to develop CHD and Alzheimer disease [62]. One PXE patient was even characterized by a homozygous ϵ2 isoform of APOE, which can be a risk factor for type III hyperlipoproteinemia in 5- 10% of ϵ2 homozygous carriers [62].

Gene expression data of array analysis displayed a strong reduction in APOE transcript levels in PXE fibroblasts. We confirmed our observations by further qPCR, whereby mRNA expression of APOE exhibited a significant increase in LPDS and under serum-free cell culture conditions, comparable to the ABCC6 expression pattern. Genetic variations in APOE isoforms between examined subjects were considered by an additional analysis of homozygous ϵ3 carriers (PXE 1/2 and Ctl 2/3; data not shown). Comparison of APOE transcript levels of these samples revealed as well significantly reduced levels in PXE patients in 10% FCS (0.25-fold) and under serum starvation (0.15-fold). Hence, mRNA expression seemed to be regulated independently of genetic polymorphisms of APOE in patients and controls, as recently shown in mononuclear cells from normolipidemic and hypercholesterolemic individuals [63]. For ABCC6-silenced cells, only a slight decrease in APOE mRNA (in 10% FCS and without FCS) was detected in comparison to scrambled siRNA negative controls. However, these differences could be due to residual ABCC6 protein content of knock-down fibroblasts or siRNA transfection method carried out with liposomes, which might have a still unknown impact on overall lipoprotein metabolism.

Increased APOE levels in fibroblasts have recently been described under serum starvation [64]. Furthermore, Ishibashi *et al.* observed increased PCSK9 expression and decreased hepatic LDLR levels in Apoe ^−/−^ and Niemann-Pick type C1 (Npc1^-/-^) double knock-out mice [65]. These data suggest dependent regulations between APOE and PCSK9, which might also be important for molecular pathomechanisms in PXE. However, in addition to its functional role in lipoprotein metabolism, regulatory properties in the extracellular matrix and intracellular calcium homeostasis were also described for APOE [66]. Measurement of mRNA expression for APOD and APOL1 also showed significantly increased levels in PXE fibroblasts in comparison to controls, mainly under serum-free conditions. Interestingly, APOD expression was described as increasing under oxidative stress as a protective cellular response [67,68], a characteristic cellular hallmark in PXE [69].

In addition to these new observations in cholesterol biosynthesis and lipoprotein metabolism in PXE fibroblasts, we found increased transcript levels of CYP27A1 in PXE fibroblasts under serum starvation. However, mRNA expression data were not confirmed by siRNA-mediated knockdown. CYP27A1 is a member of the cholesterol-hydroxylating enzymes (forming 27-hydroxycholesterol/27-OH) expressed in most body tissues, whereas circulating 27-OH is further converted into bile acids in the liver [70]. Increasing expression of CYP27A1 could be a cellular response to higher cholesterol biosynthesis rates [71], as shown here for PXE fibroblasts. However, elevated levels of side-chain hydroxylated oxysterols (27-OH) would be expected to inhibit newly synthesized cholesterol reversely, acting as a LXR ligand [70].

Cellular lipoprotein and cholesterol homeostasis is regulated by intra- and extracellular processes, including the sterol regulatory element-binding proteins (SREBPs) as essential members of transcription control [72]. SREBP2 orchestrates the induction of LDLR, PCSK9 and HMGCR in response to sterol depletion [56]. Gene expression analysis of SREBP2 showed a comparable increase under LPDS and serum-free conditions for controls and PXE fibroblasts, as well as for siRNA-transfected cells. Transcript levels of SREBF1 were increased in 10% LPDS, but were even higher in serum-free media. SREBF1 can be induced by insulin or liver X receptor (LXR) agonists [73,74] and was shown to be significantly elevated in PXE fibroblasts under serum deprivation in comparison to controls.

Induction of ABCC2 transcription was recently shown in PXE fibroblasts [18]. These ABC transporters are closely related to ABCC6 [75]. ABCC2 was shown to increase significantly under serum deprivation in PXE fibroblasts. Interestingly, reduction in ABCC3 mRNA levels in PXE fibroblasts and ABCC6-silenced cells, seem to be absolutely independent of cholesterol biosynthesis, or lipoprotein supply. Recently, Kobayashi *et al.* found that gene regulation of ABCC2 is linked to LXR-SREBP regulatory pathways [76]. These connection should also be investigated for ABCC6, using promotor analysis for the detection of possible transcriptional factor binding sites for SREBPs.

This is the first study which links ABCC6 deficiency to higher cholesterol biosynthetic rates, alterations in LDLR- PCSK9 regulation and decreased APOE mRNA expression. All findings are important atherosclerotic risk factors and should be investigated in future studies, exploring the functional role of ABCC6 in the pathogenesis of PXE and related diseases.

## Abbreviations

ABCC6: ATP-binding cassette, sub-family C, member 6; ACTB: Actin, beta; APOD: Apolipoprotein D; APOE: Apolipoprotein E; APOL1: Apolipoprotein L1; ANGPTL3: Angiopoietin-like 3; β2M: Beta-2 microglobulin; CEL: Carboxyl ester lipase; CHD: Coronary heart disease; CYP27A1: Cytochrome P450, family 27, subfamily A, polypeptide 1; CYP39A1: Cytochrome P450, family 39, subfamily A, polypeptide 1; CXCL16: Chemokine (C-X-C motif) ligand 16; DHCR7: 7-dehydrocholesterol reductase; ERK 1/2: Extracellular signal regulated kinases 1 and 2; FCS: Fetal calf serum; FDFT1: Squalene synthase; FDPS: Farnesyl diphosphate synthase; FXR: Farnesoid X Receptor; GAPDH: Glyceraldehyde-3-phosphate dehydrogenase; GGPS1: Geranylgeranyl diphosphate synthase 1; HDL: High-density lipoprotein; HDLBP: High density lipoprotein binding protein; HMGCR: 3-hydroxy-3-methylglutaryl-CoA reductase; HNF4α: Hepatocyte nuclear factor 4 alpha; LDL: Low density lipoprotein; LDLR: Low density lipoprotein receptor; LPDS: Lipoprotein-deficient serum; LRP1B: Low density lipoprotein receptor-related protein 1B; LSS: Lanosterol synthase; LXR: Liver X receptor; NPC1L1: Niemann-Pick disease, type C1, gene like 1; OLR1: Oxidized low density lipoprotein (lectin-like) receptor 1; PCSK9: Proprotein convertase subtilisin/kexin type 9; PPAR**γ**: Peroxisome proliferator-activated receptor gamma; PXE: Pseudoxanthoma elasticum; RCT: Reverse cholesterol transport; qPCR: Real-Time quantitative PCR; siNK: Small-interfering RNA negative control; siRNA: Small-interfering RNA; SREBF1: Sterol regulatory element-binding factor 1; SREBP2: Sterol regulatory element-binding protein 2; TM7SF2: Transmembrane 7 superfamily member 2; TRERF1: Transcriptional regulating factor 1; UPLC: Ultra-performance liquid chromatography; VLDL: Very low density lipoprotein.

## Competing interests

The authors declare that they have no competing interests.

## Authors’ contributions

PK performed the experiments, data analysis, interpretation and manuscript writing. JK was responsible for mass spectrometry analysis. MDR and IF helped with the experimental work. CG, CK and DH contributed to the design of the study, data interpretation and manuscript preparation. All authors read and approved the final manuscript.

## Supplementary Material

Additional file 1: Table S1 Characterization of human dermal fibroblasts derived from PXE patients and healthy controls. Click here for file

Additional file 2: Table S2 Primer sequences used for qPCR. Click here for file

Additional file 3: Table S3 RT^2^ Profiler PCR Array: Lipoprotein signaling and cholesterol metabolism. Regulated gene expression of human dermal fibroblasts of PXE patients and healthy controls (PXE/ controls) and siRNA transfected cells (siABC6/ siNK) cultivated without FCS for 24 h. Click here for file

Additional file 4: Table S4 APOE genotyping of PXE patients and healthy controls. Click here for file
