# Supplementary material for: ABCC6- a new player in cellular cholesterol and lipoprotein metabolism?
Source: Lipids Health Dis. 2014 Jul 27;13:118. doi: 10.1186/1476-511X-13-118 (PMC4124508; doi:10.1186/1476-511X-13-118)
Supplement: Additional file 1: Table S1 — Characterization of human dermal fibroblasts derived from PXE patients and healthy controls. [file 1476-511X-13-118-S1.docx]

| **Sample iD** | **Catalog ID** | **Supplier** | **Gender** | **Age**  **(years)** | **Biopsy source** | ***ABCC6* genotype^a^** | | **Genotype status** |
| --- | --- | --- | --- | --- | --- | --- | --- | --- |
|  | | | | | | | | |
| **PXE patients** | | | | | | | | |
| PXE 1 |  | * | male | 51 | Neck | c.3769_3770insC (p.L1259fsX1277) | c.3769_3770insC (p.L1259fsX1277) | hm |
| PXE 2 |  | * | male | 41 | n/a | c.1552C>T (p.R518X) | n/d | ht |
| PXE 3 |  | * | female | 45 | Armpit | c.3421C>T (p.R1141X) | c.2787+1G>T | cht |
| PXE 4 |  | * | female | 62 | Neck | c.1132C>T (p.Q378X) | c.3421C>T (p.R1141X) | cht |
|  | | | | | | | | |
| **healthy controls** | | | | | | | | |
| Ctl 1 | PH10605A | Genlantis | male | 56 | Face | - | - | wt |
| Ctl 2 | PH10605A | Genlantis | male | 45 | Face | - | - | wt |
| Ctl 3 | CC-2511 | Cambrex | female | 42 | Abdomen | - | - | wt |
| Ctl 4 | C-12302 | Promocell | female | 52 | Cheek | - | - | wt |
|  | | | | | | | | |
| **siRNA-treated fibroblasts (siNK/ siABCC6)** | | | | | | | | |
| siNK/ ABCC6_1 | PH10605A | Genlantis | male | 56 | Face | - | - | wt |
| siNK/ ABCC6_2 | PH10605A | Genlantis | male | 45 | Face | - | - | wt |
| siNK/ ABCC6_3 | CC-2511 | Cambrex | female | 42 | Abdomen | - | - | wt |
| siNK/ ABCC6_4 | C-12302 | Promocell | female | 52 | Cheek | - | - | wt |

hm, homozygote; cht, compound heterozygote; ht, heterozygote; wt, wild type; n/a, not applicable; n/d, not detected.

* fibroblasts isolated from skin biopsies (Hendig et al. 2008, *Lab. Invest*.)

^a^ Nucleotide numbering refers to the cDNA sequence with the A of the ATG translation initiation start site as nucleotide +1 (GenBank accession number NM_001171.2)
