# Supplementary material for: ABCC6- a new player in cellular cholesterol and lipoprotein metabolism?
Source: Lipids Health Dis. 2014 Jul 27;13:118. doi: 10.1186/1476-511X-13-118 (PMC4124508; doi:10.1186/1476-511X-13-118)
Supplement: Additional file 2: Table S2 — Primer sequences used for qPCR. [file 1476-511X-13-118-S2.docx]

**Table S2.**  Primer sequences used for qPCR

| **gene** | **sequence 5´- 3´** | **reference sequence** | **product size (bp)** | **annealing (°C)** | **melting (°C)** |  |
| --- | --- | --- | --- | --- | --- | --- |
| **ACTB**  *Actin, beta* | CGCGAGAAGATGACCC  ATTGCCAATGGTGATGAC | NM_001101 | 411 | 59 | 89.1 |  |
| **GAPDH**  *glyceraldehyde-3-phosphate dehydrogenase* | AGGTCGGAGTCAACGGAT  TCCTGGAAGATGGTGATG | NM_002046 | 223 | 59 | 83.0 |  |
| **ß2M**  *beta-2 microglobulin* | TGTGCTCGCGCTACTCTCTCTT  CGGATGGATGAAACCCAGACA | NM_004048 | 137 | 59 | 82.9 |  |
| **ABCC6**  *ATP-binding cassette, sub-family C, member 6* | CCTGCTGATGTACGCCTT  ACGCGAGCATTGTTCTGA | NM_001171 | 267 | 59 | 88.7 |  |
| **HMGCR**  *Hmg-CoA reductase* | AAGTTTGCCCTCAGTTCC  ACTGACATGCAGCCAAAG | NM_000859 | 179 | 59 | 83.4 |  |
| **FDPS**  *farnesyl diphosphate synthase* | TTGCTCCTCCCTCAGAATGAAC  TGCCTCCAATGGCATTGTACTC | NM_001135821 | 185 | 59 | 85.2 |  |
| **GGPS1**  *geranylgeranyl*  *diphosphate synthase* | CCAGGTAAACAAGTGAGAACCAA  CGTCGGAGTTTTGAGTT | NM_001037277 | 154 | 59 | 79.9 |  |
| **FDFT1**  *squalene synthase* | CTACAACCTGGTGCGCTTCC  GCGAAACTGCGACTGGTCTG | NM_004462 | 126 | 59 | 87.2 |  |
| **LSS**  *lanosterol synthase* | CGCCACCTATGAGACCAAG  CCGCCGACAGAACTCTAAG | NM_001001438 | 208 | 59 | 88.4 |  |
| **TM7SF2**  *transmembrane 7 superfamily member 2* | AGAGTGGCTGGGCTTGAG  GATGCGGTAAGGCACACG | NM_003273 | 276 | 59 | 89.5 |  |
| **DHCR7**  *7-dehydrocholesterol reductase* | CAATGACAGAACCGCATCTC  AGGTGACCCACAAGGTATAG | NM_001360 | 283 | 59 | 89.0 |  |
| **LDLR**  *low density lipoprotein receptor* | CGACTGCAAGGACAAATCTG  AGTCATATTCCCGGTCACAC | NM_000527 | 122 | 61 | 87.0 |  |
| **PCSK9**  *proprotein convertase subtilisin/kexin type 9* | CAGCCTGGTGGAGGTGTATC  CCTATGAGGGTGCCGCTAAC | NM_174936 | 270 | 62 | 92.2 |  |
| **SREBP2**  *sterol regulatory element binding protein 2* | CACCCAGGGCAACTCCTATTC  GCCATTGGCCGTTTGTGTC | NM_004599 | 284 | 61 | 87.9 |  |
| **gene** | **sequence 5´- 3´** | **reference sequence** | **product size (bp)** | **annealing (°C)** | **melting (°C)** |  |
| **SREBF1**  *sterol regulatory element binding transcription*  *factor 1* | AGACGGAGCCACTGTGAAG  TTGATGGAGGAGCGGTAGC | NM_001005291 | 249 | 61 | 91.1 |  |
| **APOD**  *apolipoprotein D* | AAAGCTCCAGGTCCCTTC  GATGCAGCGTCCATTCTC | NM_001647 | 224 | 59 | 89.1 |  |
| **APOE**  *apolipoprotein E* | CACAGGCAGGAAGATGAAG  TCATGGTCTCGTCCATCAG | NM_000041 | 272 | 61 | 91.4 |  |
| **APOL1**  *apolipoprotein L1* | ATTCGTGGCTGCTGCTGAACTG  TGGTGCCTTTGTGGACCTTCTG | NM_003661 | 230 | 61 | 84.1 |  |
| **CYP27A1**  *sterol 27-hydroxylase* | CAAACTCCCGGATCATAG  GACCACCTTGTACTTCTG | NM_000784 | 296 | 59 | 90.3 |  |
| **ABCC2**  *ABCC2 ATP-binding cassette C, member 2* | CACAAGCAACTGCTGAAC  TGCCAAGAGGAATGACGA | NM_000392 | 226 | 56 | 85.3 |  |
| **ABCC3**  *ABCC2 ATP-binding cassette C, member3* | GCTCCAGCTTCCTCATCA  TGGAGCACAGGAACATCA | NM_003786 | 159 | 61 | 86.0 |  |
