# Supplementary material for: ABCC6- a new player in cellular cholesterol and lipoprotein metabolism?
Source: Lipids Health Dis. 2014 Jul 27;13:118. doi: 10.1186/1476-511X-13-118 (PMC4124508; doi:10.1186/1476-511X-13-118)
Supplement: Additional file 4: Table S4 — APOE genotyping of PXE patients and healthy controls. [file 1476-511X-13-118-S4.docx]

| **Sample iD** | ***APOE* genotype^a^** | | **Genotype status^b^** |
| --- | --- | --- | --- |
|  | ***APOE* 112** | ***APOE* 158** |  |
|  | **c.334T>C, p.C112R** | **c.472C>T, p.R158C** |  |
|  | | | |
| **PXE patients** | | | |
| PXE 1 | TT | TT | ε2/ ε2 |
| PXE 2 | TT | CC | ε3/ ε3 |
| PXE 3 | TT | TC | ε2/ ε3 |
| PXE 4 | TT | CC | ε3/ ε3 |
|  | | | |
| **healthy controls** | | | |
| Ctl 1 | TC | CC | ε3/ ε4 |
| Ctl 2 | TT | CC | ε3/ ε3 |
| Ctl 3 | TT | CC | ε3/ ε3 |
| Ctl 4 | TC | CC | ε3/ ε4 |
|  | | | |
| **siRNA-treated fibroblasts (siNK/ siABCC6)** | | | |
| siNK/ ABCC6_1 | TC | CC | ε3/ ε4 |
| siNK/ ABCC6_2 | TT | CC | ε3/ ε3 |
| siNK/ ABCC6_3 | TT | CC | ε3/ ε3 |
| siNK/ ABCC6_4 | TC | CC | ε3/ ε4 |

^a^ Nucleotide numbering refers to the cDNA sequence with the A of the ATG translation initiation start site as nucleotide +1 (GenBank accession number NM_000041.2)

^b^ APOE isoforms: ε2 (cysteine^112^/ cysteine^158^), ε3 (cysteine^112^/ arginine^158^)

and ε4 (arginine^112^/ arginine158)
